# Extracorporeal Shock Wave Lithotripsy for Management of Residual Stones after Ureterolithotripsy versus Mini-Percutaneous Nephrolithotomy: A Retrospective Study

**DOI:** 10.1371/journal.pone.0067046

**Published:** 2013-06-13

**Authors:** Zhichao Huang, Xiaokun Zhao, Lei Zhang, Zhaohui Zhong, Ran Xu, Lianping Zhang

**Affiliations:** Department of Urology, Second Xiangya Hospital, Central South University, Changsha, Hunan, China; Glasgow University, United Kingdom

## Abstract

**Purpose:**

To compare the efficacy of extracorporeal shock wave lithotripsy in managing residual stones after ureterolithotripsy and mini-percutaneous nephrolithotomy.

**Materials and Methods:**

A retrospective study was carried out of 71 patients with proximal urinary tract stones (greater than 10 mm) who underwent ureterolithotripsy or mini-percutaneous nephrolithotomy at a single institution from 2009 to 2011. The 71 patients were divided into two groups: group I (n = 37) comprised patients who underwent ureterolithotripsy, and group II (n = 34) comprised patients who underwent mini-percutaneous nephrolithotomy. Clinical characteristics, stone-free rates, stone demographics, and complications were evaluated.

**Results:**

The overall stone-free rate was 90.1%. The stone-free rates in groups I and II were 97.3% and 82.4%, respectively. There was a statistically significant difference in the stone-free rates between groups I and II (P = 0.035). Neither serious intraoperative nor postoperative complications were observed. No significant difference in complications was observed between the two groups (P = 0.472).

**Conclusions:**

The results of our study suggest that extracorporeal shock wave lithotripsy is an effective and safe auxiliary procedure for managing residual stones after primary endoscopic surgery. This procedure is associated with a satisfactory stone-free rate and a low complication rate, particularly for residual stones after ureteroscopic procedures.

## Introduction

Urolithiasis has long plagued human civilization. Management of patients suffering from urinary tract calculi is considered to be a health care problem because of its high prevalence and incidence. Stone treatment has significantly evolved from open surgery to minimally invasive surgical procedures. Because of rapid progress in endoscopic technology, ureteroscopy (URS) and percutaneous nephrolithotomy (PNL) have made endoscopic procedures more effective and less invasive. However, both remain invasive procedures that require anesthesia and special equipment. PNL has a high stone-free rate (SFR) of 74% to 83% according to the American Urological Association Guidelines [1]. Nevertheless, its invasiveness cannot be ignored because of its potentially major complication rates [2]. PNL is still associated with significant complications, such as uncontrolled hemorrhage, injury to the collecting system and surrounding structures, sepsis, kidney loss, or even death [3]. In comparison, URS is a minimally invasive procedure for stone removal [4]. However, it can still be associated with complications such as urinary tract infection (UTI), ureteral avulsion, and ureteral perforation. Meanwhile, URS requires special equipment and anesthesia, which means it can only be performed in some major clinics in China.

Unfortunately, because of the limitations of endoscopic technology and instruments, residual calculi resulting from endoscopic management of proximal urinary tract calculi are usually inevitable. These residual calculi have the potential to cause ureteral obstruction and UTI, are important risk factors for stone growth and recurrence, and may even lead to progressive renal dysfunction. Several alternative treatments are available for residual calculi, such as medical expulsive therapy, extracorporeal shock wave lithotripsy (ESWL), flexible or semirigid URS, and PNL. The best treatment modalities are still controversial, but the goal of treatment is to achieve complete stone-free status as safely and quickly as possible with minimal invasiveness.

Since its introduction in the 1980s, ESWL has revolutionized stone treatment and has become the mainstay procedure for proximal urinary tract calculi [5,6]. Because of its efficacy and lack of side effects, ESWL has become the first-line treatment modality for uncomplicated intrarenal calculi of ≤ 20 mm and proximal ureteral calculi of < 10 mm [7]. Compared with other treatment modalities for proximal urinary tract calculi, such as ureterolithotripsy (URL), PNL, laparoscopic ureterolithotomy, and open surgery, ESWL has been established as the preferred treatment modality for intrarenal and some ureteral calculi because it is noninvasive, can be performed on an outpatient basis, is anesthesia-free, and is associated with rapid recovery, a low complication rate, satisfactory clinical outcome, feasible retreatment, and few contraindications [8]. Hence, ESWL should be considered as the first-choice auxiliary procedure for residual calculi, especially small residual calculi. Previous studies have identified various parameters that influence ESWL outcomes. However, to our knowledge, no study has compared the effects of ESWL on residual calculi resulting from different primary endoscopic procedures.

## Materials and Methods

We obtained approval for this study from the Institutional Review Board of The Second Xiangya Hospital, Central South University. Informed consent was obtained from all participants in our study. The informed consent was written and specified in the operative consent.

We retrospectively reviewed 71 patients with residual calculi who were treated with ESWL as an auxiliary procedure for large proximal urinary tract calculi (1cm or larger) after different endoscopic surgical procedures at a single institution between 2009 and 2011. Patient characteristics, concomitant diseases, stone demographics, therapy features, and complications were evaluated. The inclusion criterion was the presence of residual stones after a specific endoscopic surgery (URL or mini-PNL [mPNL]) for management of large proximal urinary tract calculi (1 cm or larger) by a single experienced surgeon in our clinic. All patients with residual stones were treated with ESWL as an auxiliary procedure because of a failed consecutive endoscopic procedure or because of a patient’s desire for treatment. Exclusion criteria were nonopaque residual calculi, morbid obesity, pregnancy, irreversible coagulopathy, uncontrolled UTI, arrhythmia, calcified abdominal aorta or renal aneurysm, obstruction distal to the stone, and severe cardiopulmonary disease. All patients were evaluated preoperatively with plain X-rays of the kidneys, ureters, and bladder (KUB) and intravenous urography. The stone size was measured as the cumulative longest diameter of the stone on a plain X-ray. Preoperative laboratory evaluation included a complete blood count, coagulation profile, serum biochemistry, urine culture, and urinalysis. Prophylactic preoperative antibiotics were administrated to patients with a positive urine culture according to antibiotic susceptibility test results before ESWL until the infections were controlled. The stone site was categorized as upper calyx, middle calyx, lower calyx, or renal pelvis. All patients were divided into two groups based on the primary endoscopic surgery: group I (n = 37) comprised patients who underwent ureteroscopic holmium laser or pneumatic lithotripsy, and group II (n = 34) comprised patients who underwent mPNL with holmium laser or pneumatic lithotripsy. During mPNL, 10 patients had inaccessible stones, defined as residual stones located in a relatively independent site of the collecting system such as the lower calyx or in a calyx with an acute infundibulopelvic angle, long infundibular length, or narrow infundibular width. ESWL was performed with an electrohydraulic shock wave lithotripter (HB-ESWL-VG, Haibin Medical Equipment, Co., Ltd., Zhanjiang, China). This lithotripter uses electrohydraulic waves for shock wave generation, a water cushion for coupling, a membrane for shock wave focusing, and fluoroscopy for stone localization. The aperture of the source is 176 mm. The maximal penetration depth is 130 mm. In the load range of 3 to 9 kV, the pressure at the F2 focal point increases from 20 to 50 MPa.

All patients underwent ESWL 3 months after the prior surgery to allow sufficient time for clearance. Each patient was placed in the supine position without anesthesia. All procedures were carried out under fluoroscopic guidance and generally as an outpatient procedure. The shock wave number per ESWL procedure was limited to a maximum of 3200 waves. The electric discharge voltage was escalated from 3–5 KV to 6–9 KV. The ESWL session was finished when the limited number of shocks was met, tiny fragments were the only visible stone remnants, or no visible stone was seen. No D-J stenting was needed either before or after ESWL.

Radiological confirmation of the post-ESWL stone status by KUB was performed 4 h after ESWL to assess stone fragmentation and side effects. The postoperative follow-up protocol included KUB after 2 weeks and computed tomography (CT) after 3 months. A stone-free state was defined as no identifiable stone fragments and no evidence of obstruction on radiological studies after the prior treatment. Treatment failure was defined as large fragments (stones of > 3 mm) and no evidence of fragmentation or clearance after the prior treatment. The standard protocol recommends repeating ESWL unless the former treatment is successful. In patients who required repeated ESWL, the repeated session was performed within 2 weeks after the prior procedure.

Statistical analysis was performed using SPSS 17.0 for Windows software (SPSS, Inc, Chicago, IL, USA) with the Student’s t test for continuous variables and the chi-square and Fisher’s exact tests for categorical variables. Differences resulting in a P value of <0.05 were considered statistically significant.

## Results

Our retrospective review identified 71 eligible patients, including 39 male and 32 female patients. Patient age was 18 to 74 years (mean 41 ± 14 years). The preoperative clinical data of the patients are listed in Table 1. No significant differences were observed between the two groups with respect to all variables. In 14.8% (n = 10) of patients, UTI was diagnosed, and prophylactic antibiotics were administrated preoperatively. During the study period, the total numbers of patients who underwent URL and mPNL in our clinic were 752 and 745, respectively. Accordingly, a stone-free status, defined as the absence of any fragments on KUB or CT, was achieved in 663 patients (663/752, 88.2%) among those who underwent URL and 643 patients (643/745, 86.3%) among those who underwent mPNL.

**Table 1:** Patient demographics and pretreatment characteristics.

	Group I	Group II	P
No. of patient	37	34	0.444
Gender			0.747
Male	21	18	
Female	16	16	
Mean age ± SD (Range) (year)	39±13 (18.0-68.0)	44±15 (20.0-74.0)	0.187
Stone size (mm)			0.840
5-10	11	8	
10-20	20	20	
≥20	6	6	
Mean stone size ± SD (Range) (mm)	12.86±5.10 (5.0-21.0)	13.32±5.23 (5.0-22.0)	0.710
Stone side			0.556
Right	17	18	
Left	20	16	
Stone type			0.842
Calcium-based	30	27	
Cystine-based	2	3	
Struvite-based	5	4	
Stone site			0.993
Renal pelvis	13	11	
Upper calyx	6	6	
Middle calyx	8	8	
Lower calyx	10	9	
Concomitant disease			0.986
Hypertension	5	4	
Diabetes mellitus	3	3	
Cardiovascular or cerebrovascular disease	4	3	
Positive preoperative urine culture	6	4	0.590

SD: standard deviation

### ESWL outcome

Overall, the treatment of all 71 patients required 111 ESWL sessions. Thirty-nine (54.9%) patients required only one ESWL session for complete fragmentation of stones, 24 (33.9%) required two sessions, and eight (11.2%) required three sessions. This equates to a total of 111 therapeutic sessions with a mean of 1.56 therapeutic sessions per patient. The overall SFR after 2 weeks and 3 months of treatment was 39.4% and 90.1%, respectively. Finally, five patients (7.0%) had asymptomatic, clinically insignificant residual fragments based on the 2011 European Association of Urology Guidelines on Urolithiasis [9]. However, evaluation of the SFRs in each group revealed a statistically significant difference between patients undergoing URS versus mPNL (97.3% vs 82.4%, P = 0.035) (Table 2).

**Table 2:** Treatment results for different groups.

	Group I	Group II	P
Stone-free rate (n, %)			
2 weeks	19 (51.3%)	9 (26.5%)	0.032
3 months	36 (97.3%)	28 (82.4%)	0.035
No. of sessions			0.007
Mean ± SD (range)	1.35±0.59 (1-3)	1.79±0.73 (1-3)	

SD: standard deviation

No major complications were observed among all cases. All complications are summarized in Table 3. The most common complication was renal colic, which was observed in 18 patients (25.4%), and successfully managed with antispasmodics, overhydration, and/or oral analgesics. Gross hematuria was observed in 14 patients (19.7%), and the condition spontaneously recovered without blood transfusion or hemostatic agents. A subfebrile body temperature due to preoperative UTI was detected in five patients (7.0%), who were treated with culture-specific antibiotics until their body temperature, urinalysis, and urine culture were normal. All postoperative urine culture results were consistent with the preoperative results. No pyelonephritis or sepsis was detected in any patient after treatment. Steinstrasse development was observed in six patients (8.5%), three of whom (4.2%) were cured conservatively and three of whom (4.2%) underwent retreatment.

**Table 3:** Complications for different groups.

	Group I	Group II	P
			0.472
Renal colic (%)	8 (21.6%)	10 (29.4%)	
Gross hematuria (%)	6 (16.2%)	8 (23.5%)	
Subfebrile body temperature (%)	3 (8.1%)	2 (5.9%)	
Steinstrasse (%)	2 (5.4%)	4 (11.8%)	

## Discussion

Urinary stone management has evolved over the last 30 years. Minimally invasive techniques can now be performed for urinary stones in almost all situations. ESWL treatment is generally recommended as the first-line treatment by most guidelines for intrarenal calculi of ≤ 20 mm and some ureteral calculi of < 10 mm [9,10]. ESWL shows many potential advantages over other procedures because it provides an anesthesia-free, technically less demanding, noninvasive, and effective therapeutic modality with a low rate of complications. Thus, even almost 30 years after its introduction into clinical practice, its role in the primary treatment of urinary calculi has gained widespread popularity. However, a high stone burden is cumbersome for ESWL. An increased stone burden is directly associated with a decreased SFR. To this end, urinary calculi of > 20 mm are considered to be the relative limit for ESWL [11]. Thanks to recent advancements in endoscopic technology, URS and PNL are considered to be highly effective procedures for patients with large stone burdens. Because of limitations in medical technology and conditions, residual calculi are almost inevitable postoperatively and may lead to recurrent urolithiasis or protracted UTI. However, compared with invasive procedures, the noninvasive nature and easy retreatment with ESWL have caused it to become a well-recognized auxiliary treatment for residual calculi with a small stone burden.

To the best of our knowledge, no previous study has compared ESWL for residual calculi in patients who underwent different endoscopic procedures. Residual calculi have specific features associated with differences in surgical management and patient selection. Thus, we believe that the therapeutic efficacy of ESWL for residual calculi may also vary. Our study has some important findings. We demonstrated that the SFR is directly associated with the mode of primary surgery. In our present study, the SFR in group I was 51.3% 2 weeks after treatment and 97.3% at 3 months. However, the SFR of group II was less satisfactory than that of group I (26.5% vs. 51.3%, respectively; P = 0.032 and 82.4% vs. 97.3%, respectively; P = 0.035). Usually, PNL is chosen to manage complex proximal urinary tract calculi. Because of limitations of techniques and devices, complex proximal urinary tract calculi are usually associated with a higher incidence of large or inaccessible residual calculi postoperatively. Previous studies have already demonstrated that patients with a larger stone burden had a lower SFR than did patients with smaller stones [12,13]. Hatiboglu et al. reported that among 172 patients with renal stones ranging from 3.0 to 32.0 mm (mean, 9.2 mm) who underwent ESWL, the stone size was the variable with prognostic significance (P < 0.01) and adversely affected SFR after ESWL [12]. Similar results were reported by Al-Ansari et al., who evaluated 427 patients with single or multiple renal stones (largest diameter, <30 mm) who underwent ESWL monotherapy. Their results demonstrated that stones of <10 mm had a significantly higher SFR after ESWL than stones of >10 mm [13]. During the primary mPNL in this study, 10 patients had inaccessible calculi. A stone is considered inaccessible if it is located in a relatively independent site of the collecting system (e.g., the lower calyx or a calyx with an acute infundibulopelvic angle, long infundibular length, or narrow infundibular width) and cannot be fragmented by laser or a pneumatic lithotripter during the primary endoscopic procedure. Furthermore, inaccessible residual stones usually have minimal or no natural expansion space and an insufficient stone-fluid interface, resulting in a poorer response to shock wave disintegration than a stone lying in a more expansive space [14]. Meanwhile, anatomic features of the kidney, such as an infundibulopelvic angle of > 90°, an infundibular length of > 3 cm, and an infundibular width of < 5 mm, have a direct influence on the spontaneous passage of small fragments after ESWL, inducing a lower SFR [15]. In addition, the patient’s age and gender did not affect SFR in the present series, which is coincident with the findings in other published reports [16–18].

In the present study, no serious complications, including perirenal or subcapsular hematoma, anuria, massive hematuria, acute pyelonephritis, or sepsis, were detected. The overall rate of complications after ESWL, including renal colic, gross hematuria, subfebrile body temperature, and steinstrasse, was similar to that reported in the previous literature [13,19]. Renal colic was usually associated with spontaneous stone passage, which could be successfully treated with overhydration, antispasmodics, and oral analgesics. Generally, gross hematuria occurs because of the direct effect of the procedure on the renal tissue. In our study, gross hematuria was detected in 19.7% patients, and all cases resolved spontaneously without blood transfusion. A subfebrile body temperature developed in five patients. These patients had a coexisting preoperative UTI and successfully responded to culture-specific antibiotics. No sepsis was detected in our study. Previous studies have demonstrated that the high incidence of sepsis is significantly associated with a positive urine culture and the presence of urinary tract obstruction before ESWL [20,21]. Hence, we believe that the administration of prophylactic antibiotics in patients with UTI who have undergone ESWL can effectively decrease the rate of sepsis. Salem et al. reported that steinstrasse was observed in 24.2% of 3241 consecutive adult patients who underwent ESWL and that the development of steinstrasse had a significant correlation with the stone size (P < 0.01) [19]. In our study, steinstrasse occurred in 8.4% of patients, which can be explained by the relatively small size of the residual calculi after the prior operation. Shen P et al. performed a systematic review with a meta-analysis to assess the necessity of stenting before ESWL in the management of upper urinary stones. Their results suggest that stenting induces more lower urinary tract symptoms. However, the systematic review also demonstrated significant benefits of stenting before ESWL compared with in situ ESWL in terms of steinstrasse [22]. Thus, we believe that routine pre-ESWL stenting for all patients is controversial and should be limited to specific conditions such as a large stone burden, solitary kidney, etc.

A previous study demonstrated that the existence of certain concomitant diseases, such as hypertension, diabetes mellitus, cardiovascular disease, and cerebrovascular disease, make patients more sensitive to ESWL-related complications [23]. Therefore, it is very important to carry out recommended interventions to prevent ESWL-related adverse effects. To prevent pain and procedure-related hypertension episodes, patients with hypertension were treated with an antihypertensive drug to control their blood pressure level before ESWL. During ESWL, an appropriate sedative or analgesic medication was given to patients who could not tolerate ESWL. Meanwhile, an endocrinologic consultation was routinely performed for patients with diabetes mellitus. Urinalysis and urine culture were performed before and after ESWL. In patients with a positive urine culture, appropriate antibiotics were used until the UTI was controlled. Furthermore, many patients with cardiovascular or cerebrovascular disease received anticoagulant therapy for prevention or treatment of acute episodes of cardiovascular or cerebrovascular disease. An untreated coagulation disorder is a contraindication for ESWL, because severe hematuria or renal hematoma might occur in patients with untreated coagulation disorders [24]. Therefore, all patients were specifically informed to withdraw any anticoagulative medications for 7 to 10 days before ESWL until coagulation function test results were normal. In the present study, no complications related to renal hematoma were seen, and patients with gross hematuria showed spontaneous resolution. None required a blood transfusion or surgery.

We acknowledge that our study has some potential limitations. First, this was a retrospective review of a small group of patients from a single clinic with a short-term follow-up period. Second, because it was a retrospective study, stone cultures and associated metabolic evaluations were not performed. Third, we calculated the stone burden as the cumulative longest diameter, not in two dimensions. Further well-designed studies with long-term follow-ups are recommended to confirm the present results.

In conclusion, ESWL is an effective and safe auxiliary procedure after primary endoscopic surgery with a satisfactory SFR and few complications, particularly for residual calculi after ureteroscopic procedures.
